# Functional Hydrogels for Treatment of Chronic Wounds

**DOI:** 10.3390/gels8020127

**Published:** 2022-02-17

**Authors:** Ilayda Firlar, Mine Altunbek, Colleen McCarthy, Murugan Ramalingam, Gulden Camci-Unal

**Affiliations:** 1 Biomedical Engineering and Biotechnology Program, University of Massachusetts Lowell, Lowell, MA 01854, USA; laydafrla@gmail.com; 2 Department of Chemical Engineering, University of Massachusetts Lowell, Lowell, MA 01854, USA; mine_altunbek@uml.edu (M.A.); cmmccar@bu.edu (C.M.); 3 School of Basic Medical Sciences, Chengdu University, Chengdu 610106, China; rmurug2000@gmail.com; 4 Institute of Tissue Regeneration Engineering, Dankook University, Cheonan 31116, Korea; 5 Department of Surgery, University of Massachusetts Medical School, Worcester, MA 01605, USA

**Keywords:** chronic wounds, wound healing, wound dressings, hydrogels, polymers

## Abstract

Chronic wounds severely affect 1–2% of the population in developed countries. It has been reported that nearly 6.5 million people in the United States suffer from at least one chronic wound in their lifetime. The treatment of chronic wounds is critical for maintaining the physical and mental well-being of patients and improving their quality of life. There are a host of methods for the treatment of chronic wounds, including debridement, hyperbaric oxygen therapy, ultrasound, and electromagnetic therapies, negative pressure wound therapy, skin grafts, and hydrogel dressings. Among these, hydrogel dressings represent a promising and viable choice because their tunable functional properties, such as biodegradability, adhesivity, and antimicrobial, anti-inflammatory, and pre-angiogenic bioactivities, can accelerate the healing of chronic wounds. This review summarizes the types of chronic wounds, phases of the healing process, and key therapeutic approaches. Hydrogel-based dressings are reviewed for their multifunctional properties and their advantages for the treatment of chronic wounds. Examples of commercially available hydrogel dressings are also provided to demonstrate their effectiveness over other types of wound dressings for chronic wound healing.

## 1. Introduction

### 1.1. Skin Wounds

Skin is the largest organ in the human body, and it provides a vital barrier against the outside environment. Severe cutaneous wounds or burns trigger the healing process in a well-synchronized sequence of controlled stages: hemostasis, inflammation, proliferation, and re-modeling [1,2]. In a normal wound healing process, these stages proceed in a specific order with each stage having a proper duration. The duration of the healing stages varies for different wound types [2,3]. In the next subsection, wound healing phases are described.

### 1.2. Wound Healing Phases

**Figure 1:**
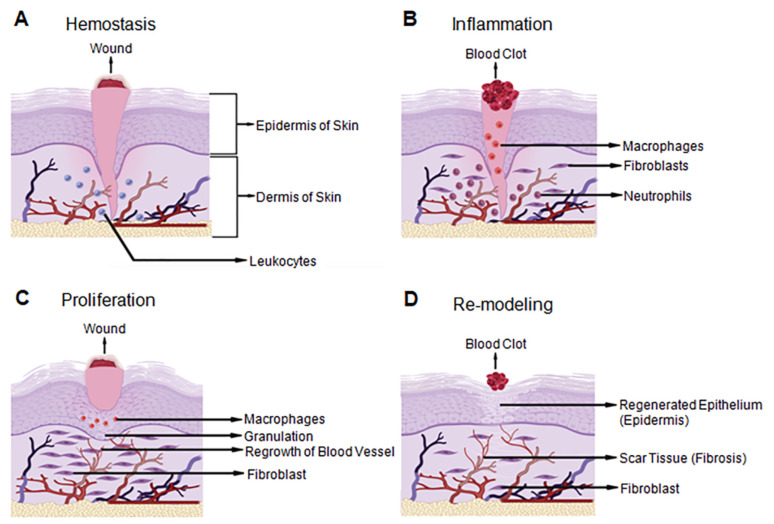
Wound healing stages demonstrated on the three layers of human skin. There are four- tightly controlled stages of wound healing in the human body: (**A**) Hemostasis is the first stage and acts as the first response when blood vessels are damaged and blood leaks into the wound area; (**B**) Inflammation is the second phase and involves vasodilation which helps to prevent infection by triggering the formation of a blood clot and cleaning the wound site with leukocytes; (**C**) Proliferation is the tissue development phase of wound healing. Granulated tissue with an extracellular matrix (ECM) composed of new connective tissue and blood vessels is formed in the presence of an appropriate amount of moisture and oxygen; (**D**) Re-modeling is the last stage and is regulated by differentiated myofibroblasts. The ECM of the wounded tissue is reconstituted similar to normal tissue. Many of the newly produced capillaries regress and restore the vascular density of the wound to normal. Created with BioRender.com accessed on 13 November 2021.

Wound healing is a natural process comprised of a series of distinct events that make up four highly regulated phases: homeostasis, inflammation, proliferation, and re-modeling (Figure 1). For a wound to heal properly, the stages should take place in the correct order and within a certain time frame. In adults, the wound healing process involves homeostasis, an inflammatory response, differentiation, multiplication, movement of mesenchymal cells, angiogenesis, brief re-epithelialization, and the synthesis, crosslinking and arrangement of collagen to give solidarity to the recuperating tissue [1,2].

Homeostasis is the initial step of wound healing. It serves as the body’s first response to damage. Blood arteries in the trauma region contract to reduce blood loss instantly. Platelets are then released along with fibrin to create a thrombus or blood clot to seal the damaged blood arteries and prevent blood loss [3].

Inflammation occurs immediately after homeostasis. The vasodilation of local capillaries helps with the transfer of leukocytes and exudates near the wound site to prevent infection throughout the wound healing process. In addition, pro-inflammatory cytokines and growth factors, including transforming growth factor beta (TGF-β), platelet-derived growth factor (PDGF), fibroblast growth factor (FGF), and epidermal growth factor (EGF), are released in the surrounding wound tissue. Meanwhile, neutrophils clear cellular debris from the wound area and kill invading microorganisms by generating reactive oxygen species (ROS) and releasing toxic proteases. Macrophages are critical to facilitate the regeneration of the skin tissue [3,4,5]. Macrophages produce cytokines in the initial stages to enhance the immune response by attracting and stimulating additional leukocytes. Moreover, macrophages induce apoptosis and clear apoptotic cells, including neutrophils. Redness and discomfort around the wound bed are commonly observed at this stage.

Proliferation is the third phase of wound healing. In this phase, granulated tissue with an extracellular matrix (ECM) composed of new connective tissue and blood vessels is formed in the presence of an appropriate amount of moisture and oxygen. The movement of T-lymphocytes (T-cells) into a wound bed plays an important role in granulated tissue formation. These T-cells secrete biomolecules, such as fibroblast growth factor 7 (FGF-7), keratinocyte growth factors (KGFs), and insulin-like growth factor-1 (IGF-1), to regulate fibroblast and keratinocyte expansion during this stage [6].

Re-modeling is the fourth and final phase of wound healing. In this stage, the ECM of the wounded tissue is reconstituted to be similar to healthy tissue. This phase is mostly regulated by differentiated myofibroblasts. Collagen, the main ECM component, is synthesized and accumulates in the granulation tissue to restore the tensile strength and elasticity that is found in normal skin tissue [7]. During this stage, many of the newly produced capillaries regress, and the vascular density of the wound is restored [3,8].

Overall, various cell types and biomolecules participate in the different wound healing phases, and their activity plays a crucial role in wound repair. The most efficient treatment approach might be chosen based on the healing stage of a specific wound. The different types of wounds are classified in the next section.

### 1.3. Classification of Skin Wounds

Skin wounds are classified based on the duration of the healing stages. If each stage of the wound healing process proceeds in a timely and organized manner, wounds will completely heal, both anatomically and functionally, within three months after the initial injury. These types of wounds are called non-chronic wounds. A delay in any of the healing stages, however, causes wounds to become chronic. This may result in a prolonged healing time or a non-healing wound, which might cause skin to lose some of its anatomical or functional properties. Delays in the wound healing processes typically occur during the inflammatory phase. The most common types of chronic wounds are diabetic ulcers, pressure ulcers, and vascular ulcers (Table 1) [9]. The effects of the chronic wound type on the healing process are discussed in the next section.

## 2. Chronic Wounds

### 2.1. Types of Chronic Wounds

Diabetic ulcers usually develop in patients with peripheral neuropathy [16]. Thus, the patients are unable to notice recurrent mild damage on their legs or feet. With this condition, an increase in glucose levels leads to a rise in the level of ROS, nitric oxide blockade, DNA alteration, protein kinase C elevation, ischemia, and inflammation around the wound. The severity can vary between patients, which may change the length of time required for wound healing. The variation in wound healing times is caused by variations in growth factor synthesis, angiogenesis, cell migration and proliferation, collagen deposition, and ECM modification by proteases [3].

Pressure ulcers are another type of chronic wound. Pressure ulcers are most seen in individuals with limited movement and neuropathies. A physical stress induced by prolonged direct pressure and shear forces on the skin can cause injury-related hypoxia and ischemia. In response, adipocytes trigger an inflammatory response. Therefore, higher concentrations of neutrophils are generally seen in patients with pressure ulcers. Necrotic tissue is also observed in these patients due to increased levels of interstitial fluid. This type of chronic wound may become more severe in patients with vascular insufficiencies [3].

There are two types of vascular ulcers: arterial insufficiency (ischemic) ulcers and blood vessel (venous) ulcers. Ischemic ulcers arise from atherosclerosis, in which prohibited perfusion flow results in ischemia and tissue necrosis. Venous ulcers are characterized by venous insufficiencies. Hence, these ulcers are frequently accompanied by edema, varicose veins, and hyper-pigmented zones. In addition, the accumulation of hemosiderin, an iron storage complex and the breakdown product of heme, is seen, which may lead to stiffness in the legs. Venous ulcers may be preceded by sensations of stiffness and discomfort in the legs and limbs similar to arterial insufficiency ulcers [3].

The stages of the wound healing process for different types of chronic wounds need to be studied to understand the mechanisms and to develop proper treatment methodologies. The next section focuses on possible treatment approaches for chronic wounds (Table 2).

### 2.2. Treatment of Chronic Wounds

#### 2.2.1. Debridement

Debridement is a well-structured, initial clinical approach to remove non-viable tissue in the wound bed for any type of wound [17]. Debridement can be performed by surgery, conventional dressing, larvae, enzyme preparation, polysaccharide beads, or hydrogels [18]. All methods show similar activity, and the debridement process must be performed by clinical specialists. If the amount of removed non-viable tissue is not sufficient, the residual non-viable tissue may cause damage to the surrounding tissues. Therefore, the debridement process may need to be repeated. However, removal of too much non-viable tissue can lead to the loss of some viable tissue and lengthen the healing time [25,26]. A single debridement process with the loss of a small number of viable cells is optimal for preserving the viability of the wound area, which helps with the reconstitution of skin tissue. In addition, patients might experience pain during and after the debridement process. The effectiveness of debridement differs from one patient to another. The overall debridement process can be a costly treatment approach [19].

#### 2.2.2. Hyperbaric Oxygen Therapy

Hyperbaric oxygen therapy is based on the use of a special oxygen chamber which exposes the patient to high pressured oxygen to increase the oxygen concentration of blood in the wound area [27]. This method is mostly preferred for diabetic ulcers [20,27]. Hyperbaric oxygen therapy has been shown to improve and shorten the wound healing process in cases where revascularization of the damaged tissue failed or was not feasible. The administration of hyperbaric oxygen therapy, however, requires costly specialized hardware. In addition, it is usually limited to diabetic wounds and pressure ulcers.

#### 2.2.3. Ultrasound and Electromagnetic Therapies

Ultrasound therapy uses sound waves to relieve pain by warming up the wound area. Side effects of ultrasound therapy include damage or burns in the endothelial tissues. These effects often occur when process parameters are not optimized according to the specific needs of a patient. Another wound treatment approach is electromagnetic therapy. This method is based on the application of weak electromagnetic waves with frequencies ranging from 30 to 70 GHz and power densities up to 10 mWcm^−2^ to relieve pain in the wound area [21]. The use of this method has been suggested for the treatment of venous and pressure ulcers and surgical incisions. Neither ultrasound nor electromagnetic therapies have been clinically proven to significantly influence the wound healing process [21,22].

#### 2.2.4. Negative Pressure Wound Therapy

Negative pressure wound therapy is also known as vacuum-assisted closure (VAC) therapy [23]. In this method, an airtight dressing, which removes air and fluid from the wound via a small tube connected to a pump, is used to cover the wound. The negative pressure can also help increase blood flow around the wound. This approach increases moisture and oxygen levels around the wound area and therefore enhances the healing process in large chronic wounds. It can be used in both primary and secondary treatment methods. VAC therapy is typically used in fully equipped healthcare facilities. The treatment procedure not only limits the mobility of the patient, but may also cause discomfort due to noise.

#### 2.2.5. Skin Grafts

Skin grafts are generally transplanted to wound sites that may not close on their own. In this method, donor skin tissue is taken from another part of the patient’s body, or a graft is produced from a human donor or synthetically [24]. This approach, however, requires the expertise of medical professionals at fully equipped health institutions. Moreover, skin grafting operations are typically not cost-effective.

#### 2.2.6. Wound Dressings

Wound dressings are used to cover wounds to provide optimum conditions for the wound healing process [28,29,30,31]. The desired characteristics of an ideal wound dressing for efficient clinical performance should be as follows: (i) maintaining moisture in the wound environment while absorbing or eliminating excess fluids and exudates, (ii) allowing gas transmission, (iii) protecting against microbial invasion, (iv) providing a barrier to protect the wound from external trauma, (v) enabling easy removal or having biodegradable properties to avoid painful removal and damage to newly formed tissue, (vi) keeping cells viable and decreasing surface necrosis, (vii) alleviating wound pain, and (viii) being cost effective. Different types of wound dressings have been explored, but their applicability changes depending on the wound features, such as wound depth and amount of exudate (Table 3). In the following section, commonly used wound dressing types and their applications are discussed.

##### Films

A film dressing is an optically transparent, thin layer of polymeric material used to provide a barrier against external contamination and damage while keeping the wound environment moist [32]. They can be prepared to have adherent, gas permeable, and antimicrobial properties. However, the removal of a film dressing can be challenging and can damage newly formed tissue. In addition, since the films cannot collect and remove fluid from a wound environment, accumulated fluid might damage newly differentiated keratinocytes. To overcome the risk of cell and tissue damage, dressings that can easily be removed from chronic wounds should be considered.

##### Gauze

Gauze dressings are known as wet-to-dry dressings [28,33]. Gauze is typically fabricated as a thin polymeric material that is transparent, elastic, gas permeable, nontoxic, biocompatible, and biodegradable. Gauze has a free and open weave structure. The weft threads are organized in pairs and crossed before and after each warp yarn. This organization keeps the gauze securely in place. Gauze dressings dry the superficial wound debris and adhere to necrotic tissues and remove them from the wound bed [33]. In addition, gauze dressings protect the wound from microbial contamination. However, tissue cooling during the transition from inflammation to proliferation phase can impede leukocyte and phagocyte function [34]. In addition, gauze can adhere to the tissue surface during drying, which might hinder tissue healing by causing hypoxia, vasoconstriction, or re-injury of the wounded area. The functionality is improved when the gauze is impregnated with materials such as petrolatum, saline, and hydrogels [35]. These modifications enable gauze to maintain moisture in the wound area, and the adverse effects are eliminated by preventing local cooling.

##### Foam Dressings

Foam dressings are strong adsorbents capable of handling the high level of exudates in chronic wounds. They insulate the exudate without adhering to the wound area, and they provide a moist environment. Foam dressings may require a secondary dressing to secure them on the wound, but they can be modified to have adhesive borders which adhere to the skin without adhesion to the wound bed [28,36]. Foam might adhere to the wound if the wound is dry or has low amounts of exudate.

##### Wound Fillers

Wound fillers are non-adherent biomaterials in the form of pastes, granules, or powders function to moist the wound bed, and absorb exudates. The application and removal of wound fillers are easy, but a secondary dressing such as gauze is required. The filler composition determines the absorption capability [37].

##### Hydrocolloid Dressings

Hydrocolloid dressings mostly create a moist and insulated environment that can protect non-infected wounds while stimulating the body’s natural enzymes to improve the quality and formation of granulated tissue [38]. However, hydrocolloid dressings also have limitations. They might over-promote the formation of granulation tissue [39] and facilitate a gel-like fluid drainage with an unpleasant smell. By the same token, it may be difficult to treat wounds around cavities using hydrocolloid dressings [40]. While this method has several advantages for wound healing, new and more comfortable strategies that are easy to implement can be used as an efficient treatment for chronic wounds.

##### Hydrogel Dressings

Hydrogel dressings are three-dimensional networks of hydrophilic polymers. They provide a moist environment in the wound site which promotes tissue regeneration by granulation and re-epithelialization [41,42]. Hydrogel dressings have been conveniently used for the treatment of chronic wounds. Compared to other chronic wound treatment strategies, the properties of hydrogel-based dressings can easily be modified. Due to their flexible and tunable properties, hydrogel dressings can obtain additional functional properties by loading cells, antibacterial, antiviral, and antifungal agents, growth factors, and biomolecules to speed up wound contraction and healing. Hydrogel dressings can be developed for specific wounds based on their size, severity, and location. In addition, these dressings can be easily applied to irregular or deep wounds due to their ability to induce in situ and cytocompatible chemical crosslinking [45,46,47,48]. In the next section, we review functional hydrogels for chronic wound healing.

## 3. Functional Hydrogels for Chronic Wound Healing

Hydrogels have great potential to present multi-functional properties. Different functional aspects, such as biocompatibility, biodegradability, adhesiveness, vascularization potential, as well as antimicrobial, anti-inflammatory, and pro-angiogenic properties, can be incorporated into hydrogels for chronic wound healing (Table 4). Biocompatibility is a vital requirement for a hydrogel to maintain tissue homeostasis by presenting a suitable matrix without damaging the local tissue during chronic wound healing. Biodegradability and the biodegradation rate of the hydrogels are other important aspects because they provide temporary template during the proliferation of fibroblasts, re-epithelialization and neovascularization, and remodeling of chronic wounds. Bioadhesivity also plays an important role for the long-term stability of the hydrogel dressings around the wound area, improving the homeostatic effect, keeping the wound moist, and absorbing the tissue exudates during healing. Since the prolonged healing of chronic wounds can increase the risk of infection which adversely affect the healing process, antimicrobial hydrogels can be useful to prevent infections. As indicated in Section 1.2, the inflammatory phase is the main reason for the delayed healing of chronic wounds. Anti-inflammatory hydrogels can shorten the wound healing period by facilitating the transition from the inflammatory to the proliferation stage. Limited oxygen and nutrient delivery to the wound area is another reason for the delayed chronic wound healing. Pro-angiogenic hydrogels can stimulate angiogenesis to deliver the required nutrients and oxygen to the wound bed to accelerate chronic wound healing. The functionality of hydrogels can be further improved by incorporating various types of drugs or therapeutic agents into the hydrogels. Drug or therapeutic agent releasing hydrogels can provide controlled and sustained delivery in response to the environmental stimuli.

Overall, hydrogel-based wound dressings offer promising solutions in broad aspects for the treatment of chronic wounds [43,44]. In the next section, the functionalities of these hydrogels are discussed in detail with recent literature examples for chronic wound healing.

### 3.1. Biodegradable Hydrogels for Chronic Wound Healing

Biodegradability is the ability of a material to decompose after interaction with a biological environment. The rate of hydrogel biodegradation is critical during the repair and regeneration of chronic wounds, especially deeper ones [65], such as third degree burns [66] and diabetic foot ulcers [67]. Ideally, the degradation rate should match the rate of new tissue formation and remodeling. The rate of biodegradability can be tailored using different strategies, including changing the crosslinking degree between polymer chains, blending/combining different types of polymers at different ratios, or introducing protease sensitive chemical functional groups [54,55,57,60,68,69]. ECM derived protein-based polymers, such as collagen and gelatin, are commonly used to generate biodegradable hydrogels [70]. These biomaterials are useful since their intrinsic cell recognition molecules are favorable for cell attachment and controlled enzymatic biodegradation during cell proliferation and tissue remodeling. Both collagen and gelatin can form hydrogels by physical crosslinking in response to temperature change. However, gelatin cannot retain the intact hydrogel structure at physiological temperatures. Cytocompatible chemical crosslinking approaches are commonly used to make gelatin-based hydrogels stable. Gelatin is often modified with methacrylate groups (GelMA) for the formation of rapid and in situ photocrosslinkable hydrogels [47,48]. Rapid degradation can be a drawback of gelatin-based hydrogels, but increasing the concentration of gelatin and the degree of crosslinking between polymer chains can help tailor the degradation behavior during the wound healing process [71,72,73,74]. Polysaccharide-based natural hydrogels, such as chitosan, alginate, dextran, and hyaluronic acid, are also widely used biodegradable materials in chronic wound healing studies [68]. These materials can enhance granulation, migration, and neoangiogenesis during wound healing, but they exhibit a lower rate of biodegradation compared to ECM-derived protein-based polymers [75]. Hydrogels formed by the mixing/blending or chemical crosslinking of proteins and polysaccharides displayed acceptable degradation behavior during wound healing [76,77]. For example, mixing methacrylated chitosan (ChMA) and gelatin (Gel) reduced the degradation behavior of chitosan depending on the extent of photocrosslinking and gelatin content [54]. In another study, a hybrid hydrogel was constituted by photocrosslinking of GelMA and hyaluronic acid (HAMA) [78]. The formation of stronger covalent bonds between GelMA and HAMA increased the hydrogel’s resistance to collagenase biodegradation compared to pristine GelMA hydrogels.

### 3.2. Bioadhesive Hydrogels for Chronic Wound Healing

Bioadhesive hydrogels facilitate the adhesion of a hydrogel dressing to a wet wound bed and can provide long-term stability. Due to their flexibility and stretchability, the presence of a hydrogel on the wound provides comfort to the patient. Compared to conventional wound dressings, such as gauze and films, bioadhesive hydrogels are biologically more compatible and can be removed more easily from the wound. The bioadhesive property can be provided to the hydrogel dressings by modification with polyphenol derived moieties, such as catechol, dopamine, gallic acid, or tannic acid [51,52,58,79,80].

**Figure 2:**
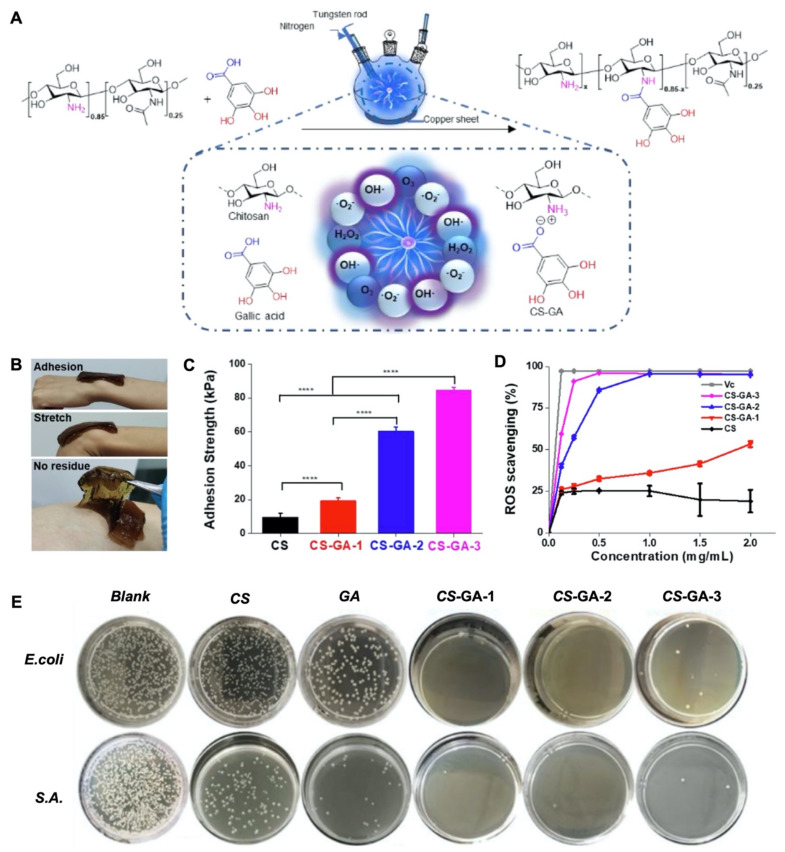
Development of bioadhesive, antioxidant, and antimicrobial multifunctional chitosan-based hydrogels for wound dressings. (**A**) Schematic representation for modification of chitosan with gallic acid (CS-GA). (**B**) Demonstration for adhesion, stretching, and removal of CS-GA from the skin surface. (**C**) Adhesion strength comparison (Student’s *t*-test, **** *p* < 0.0001). and (**D**) ROS scavenging performance based on the amount of GA grafting (**E**) Antimicrobial activity of CS, GA, and CS with different GA contents (G1 < G2 < G3) against *Escherichia coli* (*E. coli*) and *Staphylococcus aureus* (*S.A.*). Reproduced with permission [58]. Copyright 2022 Elsevier.

The degree of adhesiveness can be tailored with the extent of chemical modifications of polyphenol derived moieties to meet the demands of the particular wound type. Highly adhesive hydrogel dressings were found to be beneficial for shallow and small chronic wounds while less adhesion was preferred for deep and large chronic wounds [58]. For example, Sun et al. performed gallic acid (GA) modification to chitosan (CS-GA) (Figure 2A) with different amounts of grafting (CS-GA1 < CS-GA2 < CS-GA3) [58]. The final product CS-GA was highly biocompatible, adhesive, and stretchable (Figure 2B). The bioadhesion capacity was found to be enhanced with greater amounts of grafted GA (Figure 2C). In addition, the GA modification also enabled concentration dependent ROS scavenging activity (antioxidant) and antibacterial activity (Figure 2D,E). CS-GA hydrogels resulted in greater healing compared to gauze, gelatin sponge, and pristine CS in an in vivo full-thickness skin defect model. The more efficient wound closure with CS-GA dressings compared to the pristine CS dressings in this study was attributed to the enhanced bioadhesivity and antioxidant activity of the hydrogel dressings.

### 3.3. Antimicrobial Hydrogels for Chronic Wound Healing

Hydrogel dressings provide the first barrier against the invasion of microorganisms to the wound bed. However, due to excessive inflammation and prolonged healing, there is an increased risk of infection in chronic wounds. To address these risks, antimicrobial hydrogels have been developed with antibacterial, antiviral, or antifungal components depending on the type of infection [81].

Antibacterial hydrogels are commonly developed by incorporating antibacterial agents or inorganic materials into hydrogels [82,83]. Organic materials, such as gentamicin (GEN), vancomycin (VAN), ciprofloxacin (CIP), hematoporphyrin, fluoroquinolone, penicillin, cephalosporin, and moxifloxacin, are the most common antibacterial agents incorporated into hydrogel dressings for the treatment of chronic wounds [84,85,86,87]. These antibacterial agents act to block bacterial DNA duplication or protein synthesis. A controlled and sustained release of the antibacterial agents from the hydrogel is essential to prevent drug resistance. The release of the antibacterial agents can be controlled by modifying the stiffness, degradation, or swelling properties of hydrogel. Antibiotic release can also be controlled by modifying the hydrogels with chemical groups sensitive to environmental stimuli, such as pH or temperature [86,88].

Inorganic materials show enhanced antibacterial activity against a broad spectrum of microorganisms compared to organic antibacterial drugs. Their antibacterial activity is due to the damage that they create on bacterial cell membranes or subcellular structures [82]. Nobel metals, such as silver (Ag) and gold (Au) ions or their nanoparticles (NPs), have been utilized to add antimicrobial properties to wound dressings [82,83]. They can be directly loaded into hydrogels, or they can be stabilized into micro vesicles, such as liposomes, before being loaded into hydrogels. Their physicochemical properties, such as particle size, morphology, and surface chemistry, can be easily fine-tuned to control their antimicrobial performance in hydrogel wound dressings. Among them, Ag^+^ and Ag NPs are the most widely used because at appropriate amounts, they show high antimicrobial activity without inducing cytotoxicity in mammalian cells. The activity of Ag NPs against both Gram-positive and Gram-negative bacteria was demonstrated when they were incorporated into hydrogels [89].

Metal oxides also showed antibacterial properties when they are incorporated into hydrogels for chronic wound healing. For example, zinc oxide (ZnO), titanium dioxide (TiO_2_), and copper oxide (CuO_2_) have been reported to have antibacterial activities [90,91]. Some metal nanoparticles display antibacterial activity against only certain types of bacteria [82]. However, some of the inorganic materials might not have good cytocompatibility with mammalian cells.

There are hydrogels that possess intrinsic antibacterial properties. Chitosan, for example, is known to have inherent antibacterial activity. The electrostatic interaction between its polycationic structures with the negatively charged bacterial cell wall alters membrane permeability [92]. Upon destruction of the bacterial cell wall, the subsequent interaction of the chitosan with the bacterial intracellular components inhibits DNA replication.

Antibacterial hydrogels can also be developed by the modification of the polymers with antibacterial peptides or amphoteric compounds. These structures work similarly to the inorganic materials and chitosan as they induce physical damage to the bacterial membrane and inhibit bacterial proliferation [73,74,84].

Hydrogels can exhibit natural antiviral activity, or antiviral agents can be incorporated into hydrogels to add antiviral properties. For example, alginate-based biomaterials have been shown to have inherent antiviral activities against 17 types of viruses [93]. The mechanism of antiviral activity for alginate-based hydrogels is still uncertain, but in most of the studies, electron micrograph images showed induced viral aggregation which was attributed to the antiviral activity. Antiviral activity can also be achieved by incorporating antiviral drugs, such as entecavir, penciclovir, and ganciclovir, into hydrogels. The antiviral activity depends on the solubility, bioavailability, and activity of antiviral drugs, which can be improved by using them in combination with surfactants, polar lipids, or nanoparticles [86,94]. Some of these surfactants, lipids, polymers, and nanoparticles demonstrate antiviral activities even in their pristine forms [77,87,88]. Compared to those strategies, an interesting study for the development of antiviral hydrogels was reported by Hu et al. (2019) [95]. They used clinically available drugs that have guanine analogues in their structures. These guanine analogs formed hydrogen bonded quadruplex structures in the presence of metal ions which induced the formation of supramolecular hydrogels with inherent antiviral activity. The destruction of hydrogen bonds in response to a temperature change enabled the controlled release of antiviral drugs from the supramolecular hydrogel.

Antifungal hydrogels are crucial to prevent fungal infections in chronic wounds. Antifungal drugs are commonly immobilized in the polymer chains to obtain antifungal hydrogels. For example, amphotericin B (AmpB) is a well-known antifungal drug. The FDA has approved the use of an AmpB incorporated hydrogel, Amphogel, for chronic wounds due to high efficacy against *C. albicans* strains [96]. However, high doses of antifungal drugs encapsulated in the hydrogels might be toxic to mammalian cells in addition to the risk of the development of antimicrobial resistance. To avoid antimicrobial resistance, inherent antifungal activity can be provided by the immobilization of biocompatible antifungal peptides into hydrogels [87,97]. Liu et al. (2019) reported an interesting approach for the development of antifungal hydrogels [98]. Instead of water, plasma-activated water (PAW) was used to prepare polyacrylamide hydrogels (PAH). The long-lasting free radicals in PAW caused the PAH hydrogel to show antifungal activity. Depending on the plasma treatment time, the PAH showed stronger antifungal activity.

### 3.4. Anti-Inflammatory Hydrogels for Chronic Wound Healing

Anti-inflammatory hydrogels enhance the recruitment of macrophages to the wound bed and reduce ROS levels. These functions contribute to wound healing by allowing for the transition from the inflammatory to the proliferation stage, increasing the rate of healing, and minimizing the length of the healing period. Some hydrogels show intrinsic anti-inflammatory properties. For instance, CS and its derivatives can regulate the inflammation process by enhancing the secretion of TGF-β, PDGF, and IL-1, which helps accelerate angiogenesis, fibroblast proliferation, and collagen synthesis [92,99]. HA, which is a major component of the skin ECM, also shows inherent anti-inflammatory properties [100,101]. HA is involved in the signaling cascades that trigger the recruitment of inflammatory cells around the wound, enhance cytokine expression, and decrease inflammation to protect the tissue damage [102,103]. Anti-inflammatory properties can also be added to hydrogels by chemically attaching anti-inflammatory agents, such as phenolic compounds [78,93,94,96,98]. Those compounds can be obtained from plant extracts, honey, or antimicrobial peptides. Anti-inflammatory compounds are mostly recognized by the immune modulatory pathways to regulate inflammation and reduce ROS levels around the wound. Stimulating the body’s innate immune system by modifying the hydrogels with targeting agents can also be used to improve anti-inflammatory properties. For example, targeting the sphingosine-1-phosphate receptor could be an approach to activate inflammatory cells [104], which helps the transition from the inflammatory phase to the proliferative phase of wound healing [105,106,107,108,109,110]. Hydrogels containing bioceramics can also exhibit anti-inflammatory activity in wound healing applications [111]. For instance, the controlled release of silicon (Si) ions from bioceramic particles that were encapsulated in gelatin/PCL nanofibers reduced inflammation while increasing angiogenesis, collagen deposition, and re-epithelization [112,113,114].

Prolonged inflammation causes excessive accumulation of ROS around chronic wounds. The antioxidant capacity of cells to scavenge excessively produced ROS becomes limited. Hydrogels with antioxidative functions can facilitate the maintenance of the balance between ROS and antioxidants. Some anti-inflammatory hydrogels also exhibit antioxidative functions. For example, catechol-modified hydrogels show both anti-inflammatory and antioxidant activity [52,115]. The efficacy of the antioxidative hydrogels is shown by their activity in response to increased ROS levels. For example, Zhao et al. (2020) developed a supramolecular hydrogel composed of poly(ether urethane) (PEU) and various α-cyclodextrin (α-CD) inclusion complexes (ICs), which were crosslinked via a ROS sensitive linkage between ferrocene and β-cyclodextrin [116]. A naturally derived anti-inflammatory herb, rhein, was incorporated into this supramolecular hydrogel and was released to the wound bed upon dissolution of the hydrogel in response to increase in ROS levels. The efficacy of this stimuli responsive supramolecular hydrogel was demonstrated in a full-thickness wound model in diabetic mice. In this study, the ROS production was suppressed, and healing was successfully transitioned from the inflammatory to the proliferation stage [116].

### 3.5. Pro-Angiogenic Hydrogels for Chronic Wound Healing

Insufficient nutrient and oxygen delivery to the wound area is one of the possible reasons for delayed chronic wound healing. Stimulation of angiogenesis plays a crucial role in wound healing to deliver the necessary amount of nutrients and oxygen. Some hydrogels might have intrinsic pro-angiogenic properties, or they can be rendered pro-angiogenic by including angiogenesis stimulating factors into the hydrogels. For example, Patra et al. (2012) showed intrinsic pro-angiogenic properties of an *A. mylitta* silk fibroin (AM) hydrogel [117], whereas Leslie-Barbick et al. (2009) introduced VEGF into PEGDA hydrogels to stimulate angiogenesis [118,119]. Bioactive ions have the ability to stimulate or activate angiogenesis. For example, hot spring baths are rich with minerals that show therapeutic effects by stimulating angiogenesis under heat stimulation. Using hot spring bath treatments as inspiration, Sheng et al. (2021) prepared a composite pro-angiogenic hydrogel dressing for chronic wounds comprised of N,O-carboxymethyl chitosan (NOCS) and fayalite (ferrous and silicate ions containing bioceramics) [120]. Under mild heat stimulation through the photothermal effect, bioactive ions were released from the NOCS hydrogel to the wound bed. As demonstrated in full thickness wounds in diabetic mice models, the release of bioactive ions activated different angiogenic factors and signaling pathways, which resulted in enhanced angiogenesis and wound healing.

### 3.6. Drug or Therapeutic Agent Releasing Hydrogels for Chronic Wound Healing

Various types of drugs and therapeutic agents can be incorporated into hydrogels for efficient wound healing [64,121,122,123,124]. An important aspect is to provide their controlled and sustained release from the hydrogel. The pore sizes and stiffness of the hydrogels can influence the release rate of physically encapsulated drugs. For example, larger pore sizes can decrease the efficacy of drugs via more rapid release compared to hydrogels with smaller pore sizes. Drugs can be integrated into hydrogels using covalent bonding for controlled drug release kinetics [125].

**Figure 3:**
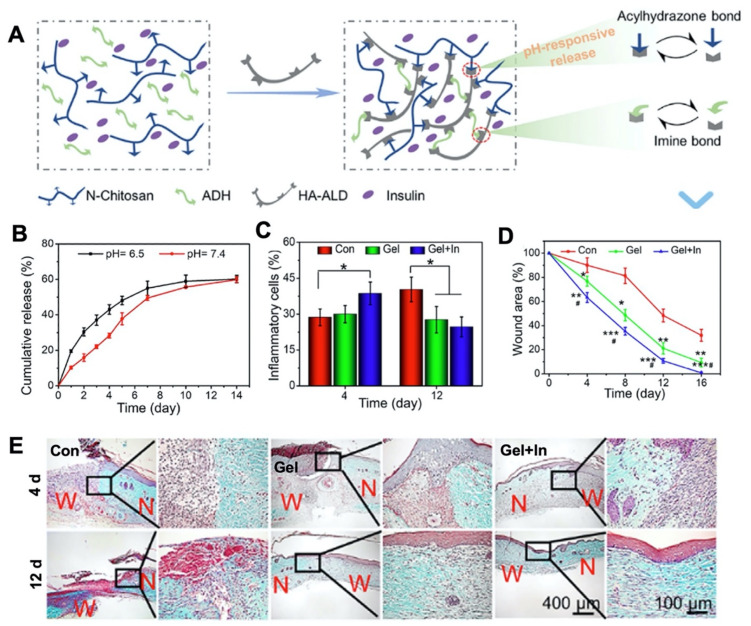
(**A**) Schematic representation for a pH-responsive chitosan based-hydrogel targeted for diabetic foot ulcer treatment. A hybrid hydrogel (Gel) was constructed between N-carboxyethyl chitosan (N-chitosan), hyaluronic acid–aldehyde (HA-ALD), and adipic acid dihydrazide (ADH). Insulin was loaded into the polymer solution and reversible dynamic bonds were provided by acylhydrazone and imine bonds (Gel+In). pH responsive properties were achieved with acylhydrazone bonds. (**B**) pH-responsive release behavior of insulin for 14 days (**C**) Quantitative analysis of the number of inflammatory cells at 4 and 12 days after the operation (Tukey’s post-hoc analysis, * *p* < 0.05) (**D**) Quantitative analysis for wound area measured on days 0, 4, 8, and 12 (Tukey’s post-hoc analysis, * *p* < 0.05, ** *p* < 0.01, *** *p* < 0.001 compared with control group; # *p* < 0.05 compared with the hydrogel group) (**E**) Masson trichrome staining to show collagen deposition on days 4 and 12 after the application of Gel and Gel+In to full thickness foot skin wounds on diabetic rats (W: Wound area, N: Normal tissue). Reproduced with permission [128]. Copyright 2021 Elsevier.

Stimuli-responsive hybrid hydrogels are useful for controlled drug delivery. These hydrogels are responsive to changes in physicochemical and biological conditions, such as pH, temperature, or glucose levels in the wound environment [59,64,126,127,128,129]. For example, a high glucose level creates an acidic environment in diabetic foot ulcers. A hybrid hydrogel (Gel) composed of N-carboxyethyl CS (N-CS), HA-aldehyde (HA-ALD), and adipic acid dihydrazide (ADH) was developed by a pH-responsive bond between acylhydrazone and imine [130]. Insulin was incorporated into the Gel (Gel+In) to be released upon elevated acidity in the wound environment (Figure 3). In response to an acidic pH, the labile acylhydrazone bond resulted in sustained insulin release. As a result, glucose levels decreased, and a full thickness wound in a rat model showed effective healing by promoted collagen deposition, and enhanced tissue formation.

In another study, a dual-responsive hydrogel against pH and ROS was designed to provide a dressing with sustained delivery of anti-inflammatory (nimesulide (NIM)) and antimicrobial (silver nanoclusters (VAN-AgNCs)) agents to the wound area [129]. This hydrogel was generated by crosslinking 3-carboxy-phenylboronic grafted gelatin with PVA. VAN-AgNCs was directly incorporated into the hydrogel while NIM was loaded in pH-sensitive micelles before being encapsulated into the hybrid hydrogels. Upon increased acidity or ROS levels in the wound environment, this hybrid hydrogel disintegrated and released the encapsulated VAN-AgNCs and micelles. The acidic environment further disintegrated the micelles to release NIM. VAN-AgNCs displayed effective antimicrobial activity while NIM exhibited an anti-inflammatory effect in both in vitro and in vivo experiments. In another study, a thermosensitive hybrid hydrogel was developed for the controlled release of MMP-9 siRNA to silence the MMP-9 gene [64]. MMP-9 is a matrix metalloproteinase displaying an important role in tissue remodeling. The excessive activation of MMP-9 causes degradation of local ECM which disrupts the cell migration and negatively affects the healing of diabetic wounds. Controlled release of MMP-9 siRNA from the thermosensitive hybrid hydrogels enabled down-regulation of the MMP-9 expression and improved the wound closure in a diabetic rat model.

**Figure 4:**
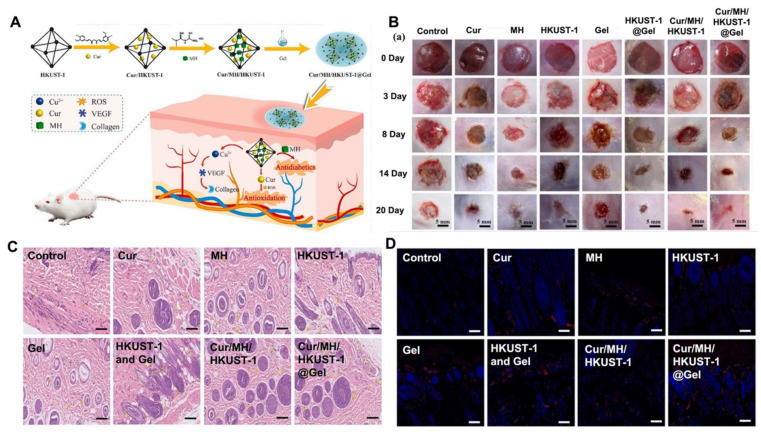
(**A**) Schematic representation of the fabrication of a drug releasing hydrogel. Metformin hydrochloride (MH) and curcumin (Cur) incorporated in a copper-based hybrid composite hydrogel Cur/MH/HKUST-1@Gel for murine diabetic wound healing. (**B**) Representative wound closure images of diabetic mice treated with Cur, MH, HKUST-1, Gel, Cur/MH/HKUST-1, and Cur/MH/HKUST-1@Gel for 20 days. (**C**) H&E staining images exhibiting tissue granulation and (**D**) Immunofluorescence staining images of CD31 to show neovascularization in mice skin tissue upon treatment with different groups. Reproduced with permission [127]. Copyright 2022 Elsevier.

In another study, a hybrid composite hydrogel was developed by integrating copper-based metal-organic frames (HKUST-1) into PLGA-PEG-PLGA (HKUST-1@Gel) for dual drug delivery around diabetic foot ulcers [127]. To regulate glucose and ROS levels around the wound, metformin hydrochloride (MH) and curcumin (Cur) were incorporated into HKUST-1 (Cur/MH/HKUST-1) before being encapsulated into the hydrogel (Cur/MH/HKUST-1@Gel). The sustained release of the Cur, MH, and copper ions exhibited synergetic effects by decreasing ROS and enhancing cell migration in vivo in a mouse model. The results also showed efficient progress in diabetic foot ulcer healing with enhanced tissue granulation and neovascularization (Figure 4).

## 4. Commercially Available Hydrogel Dressings for Chronic Wounds

Commercially available hydrogel-based wound dressings are usually in the form of amorphous gels, hydrogel sheets, hydrogel films, and hydrogel impregnated gauze (Table 5) [41]. Suprasorb^®^G (Rengsdorf, Germany) is a hydrogel film composed of approximately 70% water and 30% acrylic polymers, poly-ethylene, and phenoxyethanol. Suprasorb^®^G can be used in acute and chronic dry wounds to gently moisten the wound to prevent the formation of necrotic tissue while promoting wound healing by autolytic debridement, wound surface granulation, and epithelization. However, the Suprasorb^®^G hydrogel is limited by its ability to form a stable attachment to cavity wounds [41,131]. To overcome this issue, the addition of a secondary dressing is necessary. DermaSyn^®^ (North Bergen, NJ, USA) and AquaDerm™ (North Bergen, NJ, USA) can be used without an additional film dressing. DermaSyn^®^ is a hydrogel impregnated gauze dressing [41] while AquaDerm™ is a hydrogel dressing in sheet form [132,133]. Both dressings function similarly to Suprasorb^®^G [41,134,135]. MEDIHONEY^®^ (Princeton, NJ, USA) is another commercially available amorphous hydrogel dressing that absorbs wound exudate and transfers it into the gel for easy removal. It can be used for both acute and chronic wounds [134,136]. Similarly, ActivHeal^®^ (Winsford, Cheshire, UK) is an amorphous gel that can be efficiently applied to dry wounds to help with the debridement of necrotic tissue. Due to its efficiency, ActivHeal^®^ is commonly used against life-threatening chronic wounds [41,137]. DermaGauze™ (North Bergen, NJ, USA) is a type of gauze impregnated hydrogel. It provides adequate moisture to dry mildly exudating wounds, and it enhances autolytic debridement. Due to its secondary dressing requirement, it is a less utilized option for the treatment and management of chronic wounds [41,135]. Unlike the previous examples, the Neoheal^®^ (Ujazd, Poland) dressing is comprised of mechanically strong hydrogels that contain agar, polyethylene glycol, and polyvinylpyrrolidone. Neoheal^®^ contains approximately 90% water, debrides necrotic tissue, and promotes granulation and re-epithelization of the wound surface by enhancing angiogenesis and autolytic debridement [41,138]. Most of the other commercial hydrogel dressings for chronic wounds, such as Purilon^®^ (Humlebaek, Denmark), SOLOSITE Gel (London, UK), Restore Hydrogel (Libertyville, IL, USA), INTRASITE Gel (London, UK), NU-GEL™ (Gargrave, North Yorkshire, UK), Simpurity™ (Clarkston, MI, USA), and Woun’Dres^®^ (Humlebaek, Denmark), function similarly. They facilitate autolytic debridement and promote granulation and re-epithelization. The applications of these dressings differ depending on their material components (Table 5) [41].

## 5. Conclusions

Chronic wounds influence quality of life and present an economic burden because of prolonged treatment periods. The effects of various factors, such as pressure, ischemia, inflammation, and stress, can cause delayed healing. Different methods, such as debridement, hyperbaric oxygen therapy, ultrasound and electromagnetic therapy, negative pressure wound therapy, and skin grafts, have been used to treat chronic wounds. Each method exhibits its own advantages and disadvantages. Debridement significantly improves wound healing by shortening healing times. Hyperbaric oxygen therapy is expensive, and its use is limited to diabetic foot ulcers. Ultrasound and electromagnetic therapy can be used for venous and pressure ulcers and surgical incisions. However, these therapies might superficially damage or burn the endothelial tissues if proper parameters are not used. Negative pressure wound therapy limits patient mobility. In addition, excessive noise during treatment causes patient discomfort. Skin grafts are more expensive than other methods and require sophisticated procedures. All of the aforementioned techniques require clinical specialists with fully equipped health facilities.

Dressings are commonly used in the treatment of chronic wounds. They can be fabricated in the form of films, gauze, foams, hydrocolloids, and hydrogels. Although they are effective for preventing further damage or complications in the wound, there are some drawbacks that limit their use. Film dressings allow for the accumulation of exudates and are difficult to remove from the wound. Gauze dressings might cause discomfort and irritation on the wound. Foam dressings might adhere to the wound, making the removal challenging. Hydrocolloid dressings might cause over granulation.

Hydrogel dressings, on the other hand, are more advantageous for chronic wound treatment because they can be conveniently engineered to fulfill the specific needs of chronic wound treatment. Hydrogel dressings can promote wound healing by enhancing autolytic debridement and moisturizing the wound bed. These capabilities help healing by promoting granulation and re-epithelialization. Hydrogels can easily be removed from chronic wounds without damaging the newly differentiated keratinocytes. Furthermore, it may not be necessary to remove them from the wound due to their biodegradability. In addition, the properties of hydrogel-based dressings can be modified to enhance their adhesiveness, antimicrobial nature, vascularization ability, anti-inflammatory properties, and antioxidant features. It is anticipated that multi-functional hydrogel dressings will accelerate the development of novel products for emerging applications in the treatment of chronic wounds.

## Figures and Tables

**Table 1:** Chronic wound types.

Type of Chronic Wounds	Main Reason(s) of Occurrence	Main Symptoms	Refs.
Diabetic ulcers	Peripheral neuropathy Chronic hyperglycemia	Mild pain on legs Endothelial dysfunction and smooth muscle alterations Increased levels of glucose and ROS, nitric oxide blockade, DNA alteration, protein kinase C elevation, ischemia, and inflammation Decreased capillary size, basement membrane thickening, and arteriolar hyalinosis, growth factor synthesis, angiogenesis, cell migration and proliferation, collagen deposition, and ECM modification by proteases Necrosis of skin tissue	[3,10]
Pressure ulcers	Direct pressure and shearing forces	Tissue ischemia Epidermal, dermal, and subepidermal skin splits and mechanical stress Increased levels of neutrophiles Reperfusion hypoxia and reoxygenation Necrosis of skin tissue	[11,12]
Arterial insufficiency ulcers	Atherosclerosis	Tissue ischemia Necrosis of skin tissue	[13,14]
Blood vessel ulcers	Venous insufficiency	Sensations of stiffness and discomfort in the legs and limbs Edema Varicose veins Hyper-pigmented zones Increased levels of hemosiderin	[14,15]

**Table 2:** Currently available chronic wound treatments.

Currently Available Chronic Wound Treatments	Advantages	Disadvantages	Applications	Is It Approved for Chronic Wounds?	Does It Require a Secondary Treatment?	Refs.
Debridement	Prevent the expansion of the area of non-viable tissue while keeping the area of viable tissue stable	May cause discomfort for the patient Expensive Require a specialist Inconsistent results	All chronic wounds	Yes	Yes	[17,18,19]
Hyperbaric oxygen therapy	Increase the oxygen concentration in blood in the wound area and shortens the wound healing process	Require a specialist to apply it Expensive	All diabetic ulcers	Yes	No	[20]
Ultrasound and electromagnetic therapy	Provide pain relief to the patient	Time-consuming Expensive May have certain side effects including damage or burns on endothelial tissues	Venous and pressure ulcers Tendon injuries Surgical incisions	Yes	No	[21,22]
Negative pressure wound therapy	Increase blood flow and moisture around the wound	Require a proper health facility and medical specialist Limit the mobility of the patient Give discomfort to the patient	Certain types of venous and pressure ulcers	Yes	No	[23]
Skin grafts	Widely used Easy to apply	Only used for large wounds Require a proper health facility and a specialist to apply it Expensive Less accessible in some countries	Large chronic wounds	Yes	No	[24]

**Table 3:** Commonly used wound dressings for chronic wounds.

Wound Dressing Types	Function	Application	Advantages	Disadvantages	Refs.
Films	Surround the wound area Provide gas exchange	Shallow chronic wounds Low exuding chronic wounds Dry chronic wounds	Provide high levels of moisture Create an antibacterial environment Stabilize the wound site	No absorptive capacity May adhere to wounds	[32]
Gauze	Debridement	Shallow chronic wounds Granulated chronic wounds	Cost-efficient Widely available Thin Transparent Elastic Gas permeable Nontoxic Biocompatible Biodegradable Easily removable	Need to be frequently changed No moisture Non-selective debridement Enhance hemoglobin’s oxygen affinity May cause hypoxia, vasoconstriction, cooling, and re-injury Cause discomfort for the patient	[28,33,34,35]
Foam dressings	Absorb fluids and gel-like molecules Balance environment moisture	High exuding chronic wounds	Non-adherent Comfortable Soft Conformable May be used to prevent over-adherence	Desiccate dry chronic wounds Desiccate low exuding chronic wounds	[28,36]
Wound fillers	Moist wound area Autolytic debridement	Deep wounds Infected wounds Dry wounds Third-degree burns	Non-adherent Easy application and removal Provide high levels of moisture	May adhere to wounds	[37]
Hydrocolloid dressings	Absorb the fluids and gel-like molecules Provide autolytic debridement	Low exuding chronic wounds Non-infected chronic wounds	Provide high levels of moisture Impermeable to different molecules and compounds including water, urine, and stool Waterproof	Create an antibacterial environment Cannot be used for infected wounds Leave residues May over promote granulation tissue	[38,39,40]
Hydrogel dressings	Provide autolytic debridement Soften and loosen necrosis Rehydrate wound bed and provide moisture	Low exuding chronic wounds Dry chronic wounds Both shallow and deep chronic wounds	Provide high levels of moisture Provide pain relief May become adherent or can be used as non-adherent Provide breathable platform	May not be beneficial to use for high exuding wounds Might allow the growth of gram-negative bacteria May macerate some wounds Generally non-adherent	[41,42,43,44]

**Table 4:** Hybrid hydrogels as functional wound dressings.

Base Component	Secondary Component	Functionality	Outcome(s)	Refs.
Chitosan	Dextran-dopamine	pH-responsive controlled drug release Antibacterial activity Angiogenic activity Adhesive property	Controlled release of silver nanoparticles (AgNPs) and deferoxamine in acidic environments AgNPs release showed rapid antibacterial activity and simultaneous deferoxamine release promoted angiogenesis by enhancing the expression of hypoxia-inducible factor-1 alpha (HIF-1α) and VEGF	[49]
Chitosan	Poly(vinyl alcohol) (PVA)	Antimicrobial effect Sustained release of Ag+ and epidermal growth factor (EGF)	Enhanced re-epithelization Sufficient collagen deposition	[50]
Chitosan	Poly(d,l-lactide)-poly(ethylene glycol)-poly(d,l-lactide) (PLEL)	Thermo-sensitive Antibacterial activity Adhesive properties	Catechol modified quaternized chitosan (QCS-C) enhanced tissue adhesion Enhanced antibacterial properties Loading nano-scaled bioactive glass promoted angiogenesis by up-regulating the gene expression of VEGF and b-FGF Enhanced wound healing	[51]
Chitosan	Hyaluronic acid (HA)	Adhesive properties Anti-inflammatory activity Antioxidant effect	Catechol-containing hydrogels presented adhesion strength to the wet surfaces Supported mesenchymal stem cell growth, migration, and proliferation Protected cells against oxidative stress by controlled and sustained in situ delivery of catechol Promoted down-regulation of the pro-inflammatory cytokine IL-1β	[52]
Chitosan	Alginate and Polydeoxyribonucleotide (PDRN)-loaded CaCO_3_ nanoparticle (PCNP)	Controlled gene delivery Anti-inflammatory Pro-angiogenic	PCNP improved the in situ delivery efficacy of PDRN Accelerated proliferation of fibroblasts Increased amount of collagen fiber deposition, blood vessel formation, and cell attachments Accelerated wound healing	[53]
Chitosan	Gelatin	Biodegradable Biocompatible	Uniformly interconnected 3D porous structures Tailored degree of swelling and degradation behavior by increasing photocrosslinking and increasing gelatin concentration	[54]
Chitosan	Oxidized HA-graft-aniline tetramer (OHA-AT)	Biodegradable Antibacterial activity Electroactive Antioxidant effect Neovascularization	Accelerated wound healing by increasing granulation tissue thickness, collagen disposition and angiogenesis Amoxicillin loading added effective antibacterial activity	[55]
Chitosan	Arginine-based poly(ester urea urethane) (Arg-PEUU)	Anti-inflammatory activity Antibacterial activity Biodegradable	Methacrylate-modified chitosan (CS-GMA) and Arg-PEUU hybrid hydrogels exhibited an excellent antibacterial activity Hybrid hydrogel showed high water content, a three-dimensional microporous network structure, cytocompatibility, and enzymatic biodegradability	[56]
Chitosan	Decellularized extracellular matrix (dECM) and Gelatin	Antibacterial Biocompatible	Interconnected pore structure with high porosity promoted cell growth Degradation rate matched with the new tissue formation in skin tissue engineering Antibacterial activity Maintained the moisture and nutrition balance	[57]
Chitosan	Gallic acid (GA)	Adhesive property Antibacterial activity Homeostasis properties	Exhibited favorable antioxidant properties, high biocompatibility, and haemocompatibility High capacity of homeostasis and promoted wound healing	[58]
Chitosan	PVA and PEG	pH/glucose-triggered drug release Anti-inflammatory Neovascularization	pH and glucose-responsive drug delivery activity Enhanced wound closure rate, inflammatory infiltrate, neovascularization, and collagen deposition with the incorporation of the insulin/L929 into the hydrogel in vivo diabetic wounds	[59]
Gelatin	Lipopeptide-surfactin (SF)	Angiogenic activity Anti-inflammatory	GelMA-SF hydrogels promoted diabetic wound healing via regulating macrophage polarization and promoting angiogenesis	[60]
Agar	Fumaric acid (FA) and incorporated Ag NPs	Antibacterial Biodegradable	Controlled Ag ion release and microbial growth inhibition Accelerated healing rate with promising epithelialization, angiogenesis, and less lipid peroxidation Organized collagen deposition	[61]
Dextran	Poly(ethylene glycol) diacrylate (PEGDA)	Biodegradable Neovascularization Pro-angiogenic	Slower degradation of the dextran hydrogel with the high content of nondegradable PEGDA and higher cross-linking density Dextran hydrogel promoted rapid, efficient, and functional neovascularization without the addition of growth factors or cytokines Neutrophil cell infiltration expedited hydrogel degradation, which lead to vascular cell infiltration. Complete skin regeneration	[62]
Dextran	PEG	Controlled release of immune stimulatory cargo proteins Anti-inflammatory	Controlled release of cargo proteins Improved retention and effectiveness of an immune-stimulatory protein in the wound environment	[63]
Methylcellulose	Pluronic F-127	Thermosensitive controlled release of MMP-9 siRNA Gene delivery	Down-regulation of MMP-9 expression Enhanced diabetic wound healing	[64]

**Table 5:** Commercially available hydrogel wound dressings for chronic wound healing.

Commercial Name	Manufacturer	Contents	Chronic Wound Applications	Secondary Wound Dressing Requirement	Refs.
ActivHeal^®^	Advanced Medical Solutions Ltd.	Primary wound dressing with 85% water	Cavity wounds Pressure ulcers Diabetic foot ulcers Leg ulcers	No	[41,137]
AquaDerm™	DermaRite Industries	2-Acrylamido-2 methyl-1 propanesulfonic acid sodium Propylene Glycol Polyethylene glycol dimethacrylate 2-Hydroxy-2 methylpropiophenone 38–55% water	Minor burns Pressure ulcers Radiation Tissue damage related chronic wounds	No	[132,133]
DermaGauze™	DermaRite Industries	Impregnated gauze with acrylate polymer	Both partial and full thickness chronic wounds	Yes	[41,135]
DermaSyn^®^	DermaRite Industries	Vitamin E Primary wound dressing	Both partial and full thickness chronic wounds	No	[41]
INTRASITE Gel	Smith and Nephew	Carboxymethyl cellulose Propylene glycol	Diabetic foot ulcers Surgical incisions Leg ulcers Venous ulcers	No	[41]
MEDIHONEY^®^ (adhesive hydrogel sheet)	Integra LifeSciences Cbrp.	Adhesive hydrogel sheet Glucose oxidase and Leptospermum compounds	Pressure ulcers First- and second-degree partial-thickness burns Surgical incisions Diabetic foot ulcers Low exuding chronic wounds Venous ulcers	No	[134,136]
Neoheal^®^ Hydrogel	Kikgel	PEG Polyvinylopyrrolidone Agar Electron beams for crosslinkage 90% water	Low exuding scabs Abrasions Dry scabs First-, second-, and third-degree burns and ulcers Bed sores and chronic wounds	No	[41,138]
NU-GEL™	Systagenix	Sodium alginate Primary wound dressing	Diabetic foot ulcers Leg ulcers Venous ulcers	No	[41]
Purilon^®^	Coloplast	Calcium alginate Sodium carboxymethyl cellulose N/A% purified water	Pressure ulcers First and second-degree burns Non-infected diabetic foot ulcers Leg ulcers	No	[41]
Restore Hydrogel	Hollister Incorporated	Gauze pad Hyaluronic acid	Both partial and full thickness chronic wounds	No	[41]
Simpurity™ Hydrogel	Safe n’Simple	Absorbent sheets Acrylate Polyvinyl alcohol Polyethylene oxide Polyurethane Purified water	First- and second-degree partial-thickness burns Low exuding scabs and chronic wounds Dry scabs and chronic wounds	No	[41]
SOLOSITE Gel	Smith and Nephew	Carboxymethyl cellulose Glycerol Sodium salt At least 60% water	Diabetic foot ulcers Surgical incisions Leg ulcers Venous ulcers Minor partial-thickness burns Skin tears	No	[41,139]
Suprasorb^®^ G	Lohmann & Rauscher Global	Acrylic polymers Polyethylene Phenoxyethanol 70% water	Dry chronic wounds Low exuding chronic wounds Pressure ulcer Lower leg ulcers First and second-degree burns	Yes	[41,135,137]
Woun’ Dres^®^	Coloplast	Carbomer Collogen Other polymers Linkage molecules (N/A)	Low exuding chronic wounds Dry chronic wounds	No	[41]
